# Roles of Autophagy and Autophagy-Related Proteins in Antifungal Immunity

**DOI:** 10.3389/fimmu.2016.00047

**Published:** 2016-02-18

**Authors:** Masashi Kanayama, Mari L. Shinohara

**Affiliations:** ^1^Department of Immunology, Duke University School of Medicine, Durham, NC, USA; ^2^Department of Molecular Genetics and Microbiology, Duke University School of Medicine, Durham, NC, USA

**Keywords:** autophagy, LC3-associated phagocytosis, fungal infection, phagocytosis, macrophages, *Candida*, *Cryptococcus*, *Aspergillus*

## Abstract

Autophagy was initially characterized as a process to digest cellular components, including damaged cell organelles or unused proteins. However, later studies showed that autophagy plays an important role to protect hosts from microbial infections. Accumulating evidences showed the contribution of autophagy itself and autophagy-related proteins (ATGs) in the clearance of bacteria, virus, and parasites. A number of studies also revealed the molecular mechanisms by which autophagy is initiated and developed. Furthermore, it is now understood that some ATGs are shared between two distinct processes; autophagy and LC3-associated phagocytosis (LAP). Thus, our understanding on autophagy has been greatly enhanced in the last decade. By contrast, roles of autophagy and ATGs in fungal infections are still elusive relative to those in bacterial and viral infections. Based on limited numbers of reports, ATG-mediated host responses appear to significantly vary depending on invading fungal species. In this review, we discuss how autophagy and ATGs are involved in antifungal immune responses based on recent discoveries.

## Host Immunity Against Fungi

Pathogenic fungi, such as *Cryptococcus, Candida, Aspergillus*, and *Pneumocystis*, are considered to be four major genera of human fungal pathogens (1). A major host risk factor for development of these fungal infections (mycosis) is immunological incompetence, as they are more commonly invasive in patients with immunodeficient disorders and those who receive immunosuppressive treatments (2, 3).

The innate immune system plays a critical role in host protection against fungi. Specific defects in innate immunity, such as neutropenia or functional deficiency of NADPH oxidase, allow hosts to develop invasive aspergillosis, candidiasis, and other mycosis (3, 4). For hosts to initiate antifungal immune responses, fungi have to be detected by pattern recognition receptors (PRRs). Neutrophils and inflammatory macrophages are the first inflammatory cells recruited to the site of infection and the major killers of fungi during an early stage of infection. Dendritic cells (DCs) serve an important role as professional antigen-presenting cells (APCs) to connect innate and adaptive immunity by presenting fungal antigens to prime naïve T cells. As seen in all the microbial infections, coordinating innate immunity is the first step to protect hosts from fungal pathogens.

Defects in the adaptive immune systems are also well-known risk factors in fungal infections. Once primed by DCs, T cells activate and produce inflammatory cytokines, which further recruit innate immune cells to infected sites and facilitate phagocytosis (5, 6). B cells are also involved in fungal clearance (7, 8) by producing antibodies to opsonize fungal spores, i.e., antibodies binds spores and facilitate phagocytosis through stimulating Fc Receptor on phagocytes (9, 10).

In this review, we discuss on autophagy and autophagy-related processes in host cells during fungal infections as a part of the immune responses described above.

## Autophagy

Autophagy is a highly conserved cellular process in eukaryotes to maintain cellular homeostasis by supporting cell survival and regulating inflammation. Autophagy degrades unnecessary or dysfunctional intracellular components, such as abnormal proteins, old organelles, and pathogens, and has been widely studied in various immune cells, including T cell, B cell, macrophages, DCs, and neutrophils. Autophagy eliminates mitochondoria (mitophagy), lipid droplets (lipophagy), ribosomes (ribophagy), protein aggregates (aggrephagy), and intracellular microbes (xenophagy) (11, 12). Multiple roles for autophagy in host defense responses against microbial infections and inflammation have been reported. During a process of autophagy, a spherical double-membrane structure, termed autophagosome, is formed within a cell. A number of autophagy-related proteins (ATGs), together with other proteins, are involved in the process to form autophagosomes; starting from the formation of the autophagy initiation complex to elongating autophagosome membranes. After elongation, the membrane closes and autophagosome formation is completed. [Detailed molecular information on these steps can be found in excellent review articles (13, 14)].

It has been reported that ATGs are associated with human autoimmune disorders, cancer, and various infectious diseases (15). Single-nucleotide polymorphisms (SNPs) in *ATG16L* and *Immunity-related GTPase family M member* (*IRGM*) genes are known to increase the risk of Crohn’s disease (16, 17). A SNP of *IRGM* is also associated with susceptibility against *Mycobacterium tuberculosis* infection (18, 19), and SNPs in *ATG5* are associated with risk of systemic lupus erythematosus (20). *UV radiation resistance-associated gene* (*UVRAG*) encoding a promoter of the autophagy pathway, is monoallelically mutated at a high frequency in human colon cancer (21). Recent study demonstrated that *Atg5* in neutrophils protects mice from *M. tuberculosis* infection in autophagy-independent manner (22). Thus, autophagy and autophagy-related genes are suggested to be involved in pathogenesis of wide variety of human diseases.

Recent mechanistic studies have shown that autophagy plays an immunomodulatory role in both innate and adaptive immune responses by selectively targeting signal molecules. For example, autophagy inhibits inflammasome activation in macrophages by degrading inflammasome assemblies as well as reactive oxygen species (ROS)-producing mitochondria, which trigger activation of the NLRP3 inflammasome (23, 24). Autophagy is also known to be required for neutrophil extracellular trap formation (NETosis) (25–27) and immunological training induced by BCG or β-glucan in monocytes (28). In T cells, autophagy suppresses T cell receptor-mediated signaling by degrading BCL10, a downstream molecule of the T cell receptor (29). Autophagy also enhances memory B cell responses (30, 31). Collectively, these findings implicate autophagy in preventing excessive inflammation and protecting the host from collateral damage.

## LAP and Autophagy

LAP shares some common mechanisms and functions to autophagy; and it has been often difficult to separate LAP and autophagy. For example, LC3 staining cannot differentiate LAP from autophagy, because LC3-associated membranes are formed during both processes. LAP formation requires autophagic proteins, such as ATG5, ATG7, and LC3; therefore, mice or cells lacking one of these proteins cannot undergo both autophagy and LAP (32). Initiation of autophagy and LAP also require ROS and phosphatidylinositol 3-phosphate synthesis (33, 34); although ROS-independent LAP formation was reported in epithelial cells (35), suggesting cell type-specific signaling requirements to induce autophagy or LAP. It is possible that autophagy in non-hematopoietic cells is involved in induction of host antifungal responses. Similarly to autophagy’s contribution to microbial clearance by digesting intracellular pathogens, LAP also plays a role in pathogen clearance (36, 37). Despite the similarities, LAP is intrinsically distinct from autophagy in forming the LAPosome with a single membrane structure (32, 38), the requirement of Rubicon and NOX2 (33), and not requiring the autophagy pre-initiation complex comprised of ULK1/2, FIP200, and ATG13 (33, 39, 40).

A very recent article demonstrated that LAP is differentiated from autophagy using Rubicon-deficient mice (autophagy is intact in the mice) (33). Here, we would like to note that previously published studies using *Atg5-* or *Atg7*-deficient mice or cells might reflect impacts of impaired LAP as well as autophagy, though most of them have been published as “autophagy” studies. Therefore, it would be prudent to consider the possible involvement of LAP in previous studies, depending on an experimental condition. In this review, we use “LC3-associated cargoes” for autophagosomes and LAPosomes if responses in referred articles have not identified as autophagy or LAP.

## Roles of Autophagy-Related Proteins in Fungal Infections

The role of autophagy in antifungal immunity was strongly suggested both in mammals and plants (41). Here, we focus on autophagy in mammalian cells. Autophagy can be induced directly by signaling from fungal-sensing PRRs, and also indirectly by pro-inflammatory cytokines, including TNF-α, IL-6, IL-1, and IFN-γ (42) during fungal infections. In contrast, spores of *Aspergillus fumigatus* induce LAP based on the study using Rubicon-deficient mice (33). LAP is also induced by zymosan, a cell-wall component from *Saccharomyces cerevisiae* (33). Therefore, it is possible that other fungi induce LAP. In the following subsections and Table 1, we focus on three major fungal pathogens: *Candida albicans, Cryptococcus neoformans*, and *A. fumigatus* to discuss impacts of ATGs on host antifungal responses and pathogenesis of fungal infections.

**Table 1:** **Impacts of autophagy and LAP on antifungal immunity**.

	Approaches	Findings	Reference
*A. fumigatus*	*• Rubicon-*, *Cybb*-, *Atg4b*-, or *Ulk1*-deficient mice *• Atg3^fl/fl^LysM*-Cre mice, *Atg5^fl/fl^LysM*-Cre mice, *Atg7^fl/fl^LysM*-Cre mice, *Atg12^fl/fl^LysM*-Cre mice, *Atg14^fl/fl^LysM*-Cre mice, *Becn1^fl/fl^LysM*-Cre mice, *Rb1cc1^fl/fl^LysM*-Cre mice • shRNA for Rubicon • 3-MA • Rapamycin	*• A. fumigatus* phagocytosed by macrophages induces LAP formation • LAP, but not canonical autophagy, is required for clearance of *A. fumigatus in vitro* and *in vivo*. • Lack of LAP enhances fungi-induced pulmonary inflammation and granulomas • SNP in *Atg16L* does not affect LAP formation	(33)
	• 3-MA • Chloroquine	• Autophagy suppresses inflammasome-mediated inflammation during *A. fumigatus* infection • IL-1R blockade restores autophagy and suppresses fungal growth in CGD mice.	(58)
*C. neoformans*	• RNAi screening • siRNA for *Atg2a*, *Atg5*, *Atg9*, *Atg12*, and *Map1lc3a* (Coding LC3) • 3-MA	• Knockdown of *Atg5*, *Atg9a*, and *Atg12* decreases phagocytosis of *C. neoformans* by macrophages • Knockdown of *Atg2a*, *Atg5*, *Atg9a*, *Atg12*, and LC3 decreases fungal replication and escape of *C. neoformans* • Autophagy inhibition by 3-MA reduces phagocytosis, fungal replication, and escape of *C. neoformans*	(54)
	• shRNA for *Atg5* *• Atg5^fl/fl^LysM*-Cre mice	• Phagocytosed *C. neoformans* are surrounded by LAP in macrophages *• Atg5*-knock down by shRNA does not affect phagocytosis *• Atg5*-knock down by shRNA or *Atg5*-knock-out decreases fungicidal activity in macrophages	(50)
*C. albicans*	• shRNA for *Atg5* *• Atg5^fl/fl^LysM*-Cre mice	• Phagocytosed *C. albicans* are surrounded by LC3 in macrophages *• Atg5*-knockdown decreases phagocytosis and fungicidal activity of macrophages *in vitro* • Lack of ATG5 in myeloid cells enhances susceptibility of mice against systemic *C. albicans* infection	(50)
	*• Atg7^fl/fl^LysM*-Cre mice • 3-MA • Cohort study of candidemia patients	• Lack of ATG7 in myeloid cells does not impact on susceptibility of mice against systemic *C. albicans* infection • Autophagy inhibition by 3-MA does not affect phagocytosis and fungicidal activity against *C. albicans* in human monocytes • SNP in autophagy-related genes does not associate with incidence of candidemia	(44)
	*• Atg7^fl/fl^LysM*-Cre mice • 3-MA • Rapamycin	*• C. albicans* induces autophagy in macrophages enhances susceptibility against systemic *C. albicans* infection • Phagocytosed *C. albicans* are not surrounded by LC3 • Autophagy does not affect phagocytosis and fungicidal activity of macrophages and neutrophils • Autophagic sequestration of A20 enhances NFκB-mediated chemokine production in tissue-resident macrophages and increases neutrophil recruitment to infected site • Lack of autophagy in myeloid cells	(43)
	*• In vivo* imaging using zebra fish	• Very few LC3^+^ phagosome contain *C. albicans in vivo*	(46)
	• LC3-deficient BMMs	• Dectin-1-induced signaling triggers LAP formation in macrophages • HK *C. albicans* induces LAP in macrophages • Live *C. albicans* induces modest level of LAP in macrophages • LC3-deficiency decreases fungicidal activity of macrophages against *C. albicans*	(45)

### *Candida* *albicans*

Several studies have described roles of “autophagy” (or possibly LAP) during Candida infection. Candida spores, both live and heat-killed, are potent inducers of LC3 puncta formation and conversion of LC3-I to LC3-II (43–45). However, it appears that live Candida spores are not good at recruiting LC3 around internalized spores (43, 45, 46). These results suggest that live Candida can induce autophagy (and/or LAP), but direct clearance of *Candida* spores within LC3-associated cargoes is not likely to occur. A study by Vyas and colleagues showed that heat-killed *C. albicans* (HKCA) recruited clear LC3-associated cargoes around spores by *in vitro* observation using the RAW264.7 mouse macrophage cell line. By contrast, when live *C. albicans* was internalized, LC3-associated cargoes around spores were not very clear (45). The result is consistent with an *in vivo* study using zebra fish, in which live *C. albicans* rarely recruited significant levels of LC3 (46). We also could not detect LC3 signal surrounding live *C. albicans* in both primary macrophages and in a macrophage cell line (43). This finding was unexpected, because we speculated that Candida spores were engulfed in LC3-positve cargoes and directly killed by xenophagy or LAP. The expectation came from previous reports showing successful recruitment of LC3 to zymosan particles (33) and around β-glucan-coated polystyrene beads (45, 47). However, it is possible that live Candida spores do not expose enough β-glucan on their cell surface, while HKCA spores do (45, 48, 49). Collectively, these studies suggested that direct killing of Candida spores within LC3-associated cargoes is not very likely.

Despite the unexpectedly poor engulfment of live Candida in LC3-associated cargoes, studies have shown that autophagy (or LAP) protects hosts from Candida infections. Lack of ATG5 or ATG7 in myeloid cells decreased resistance against systemic *C. albicans* infection (43, 50). The protective role of autophagy (or LAP) in *Candida* infection is partly attributed to enhanced fungicidal activity in host myeloid cells, such as expression of ROS and efficiency of phagocytosis (50). Here, we should mention that host protection by autophagy or LAP during *Candida* infection might not be always apparent. Smeekens et al. reported no difference in survival between wild-type and *Atg7* conditional knock-out myeloid cells *(Atg7* CKO), as well as no difference in phagocytosis and killing of *Candida* (44). Although we found that *Atg7* CKO mice are more sensitive to systemic *Candida* infection, we also found no difference in phagocytosis and killing of *Candida* with or without ATG7 (43). Reasons for the discrepancy may be experimental conditions and differential strain usage of *C. albicans*. For example, Smeekens et al. used a different strain of *C. albicans* (MYA-3573) from others (SC-5314, 18804) (43, 45, 50). We used strain 18804 and found that ATG7 in myeloid cells plays a protective role in hosts without enhancing phagocytosis and killing of Candida (43). Same host responses cannot be expected when Candida strains are different. For example, published articles suggested distinct dectin-1 detection towards two different *Candida* strains, SC-5314 and 18804 (51, 52). Taken together, multiple studies suggested the involvement of ATG5 and ATG7 in enhancing resistance to Candida infection.

As shown in human studies, despite moderate influences on pro-inflammatory cytokine production, autophagy genes *ATG16L1* and *IRGM* have a minor impact on the susceptibility to both mucosal and systemic *Candida* infections (53). Other genes were investigated, such as *ATG10, ATG16L2, ATG2A, ATG2B, ATG5*, and *ATG9B*, but a clear correlation between SNPs of the genes and susceptibility to candidemia was not found (44). It was reported that an ATG16L human SNP mutant protein (T316A) expressed in mice decreases starvation-induced autophagy to 50% with no influence on zymosan-induced LAP (33). Nevertheless, impacts of these SNPs on autophagy and LAP in humans are still elusive. Therefore, further studies are needed to understand the consequence of autophagy on host immunity against Candida in humans.

### *Cryptococcus* *neoformans*

*Cryptococcus neoformans* is an opportunistic fungus. Cryptococcal yeasts are encapsulated in polysaccharides and, thus, can evade immune detection by hosts. Interestingly, host autophagy supports intracellular survival and dissemination of *C. neoformans* (54). The report demonstrated that ATGs (ATG5, ATG9a, and ATG12) are engaged, but not required, in phagocytosing *C. neoformans* by RAW264.7 macrophages, and the proteins are recruited to the vicinity of vacuoles containing *C. neoformans* (54). At a later time point (15 h after infection), ATG2a, ATG5, ATG9a, ATG12, and LC3 enhance intracellular replication and escape of *C. neoformans* from vacuoles in macrophages (54). Indeed, pharmacological inhibition of autophagy by 3-MA reduced levels of Cryptococcus infection (54). Another article reported LC3 recruitment to internalized cryptococcal spores; and mouse survival from Cryptococcus infection was not altered by *Atg5* CKO in myeloid cells (50). Nevertheless, the CKO mice exhibited reduced lung fungal burdens and protein expression of MIP-1α (CCL3), IP-10 (CXCL10), as well as Type-2 cytokines IL-4 and IL-13 (50). Expression of IFN-γ and IL-17 did not appear to be altered (50). Therefore, further studies are awaited to better understand the impact and roles of autophagy (and LAP) in Cryptococcus infection.

### *Aspergillus* *fumigatus*

LC3-II recruitment in *A. fumigatus* phagosomes was reported (55). A recent study using Rubicon-deficient mice clarified that LC3 recruitment and clearance of *A. fumigatus* are mediated by LAP, but not by autophagy (33). LAP protects mice from pulmonary aspergillosis by suppressing expression of pro-inflammatory cytokine genes and granuloma formation in the lung (33). LAP also stabilizes the NOX2 NADPH oxidase complex to produce ROS. Macrophages deficient for NOX2 failed to translocate LC3 to *Aspergillus*-containing phagosomes, as well as macrophages deficient for Beclin1, Rubicon, and ATG7 failed to do so (33). It was reported that patients with chronic granulomatous disease (CGD), caused by genetic defects in the NADPH oxidase, do not recruit LC3 around the *Aspergillus-*containing phagosomes (55). CGD patients indeed show an increased susceptibility to aspergillosis (56, 57). In addition, corticosteroid blocks recruitment of ATG (55), and its treatment is considered to be a risk factor for invasive aspergillosis (57). Another report demonstrated that IL-1R blockade protects hosts from invasive aspergillosis by increasing LC3 recruitment to *Aspergillus*-containing phagosomes and inhibiting fungal growth (58). In summary, LC3-associated cargoes appear to play a critical role in clearance of *Aspergillus* by host cells.

## Immunomodulation by Autophagy during Fungal Infection

### Regulation of Inflammasome-Mediated Immune Responses

Recent studies showed that autophagy controls immune responses during fungal infections. Autophagosomes are known to sequester assembled NLRP3 inflammasome complexes (23, 24), which are crucial for host protection against *C. albicans* and *A. fumigatus* infections (59–61). Another study showed that pharmacological inhibition of autophagy by 3-MA or chloroquine enhances inflammasome activation and inflammation in mice during *A. fumigatus* infection (58). Increased inflammasome activity was reported in monocytes and macrophages from CGD patients (58, 62–64). Therefore, autophagy (or LAP) may protect hosts from collateral damage by inflammation through downregulating NLRP3 inflammasome, which is activated by fungal infections.

### NFκB-Mediated Immune Responses

**Figure 1:**
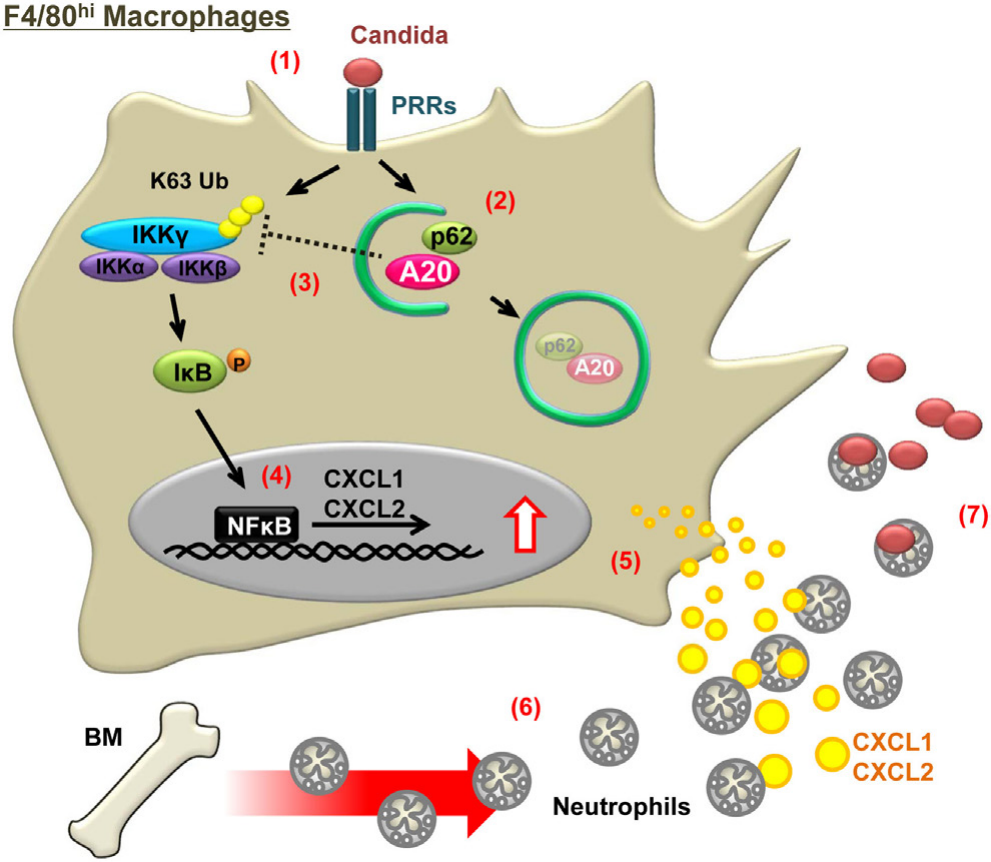
**Schematic illustration of a mechanism in which autophagy enhances antifungal immune responses by sequestering A20**. Numbers in the figure indicate steps from fungal detection to killing by hosts and correspond to the following events: (1) detection of *C. albicans* by PRRs, such as dectin-1 and TLR2, expressed on F4/80^hi^ tissue-resident macrophages; (2) autophagy induction and sequestration of A20 in autophagosomes, and A20 delivery to autophagosomes by p62; (3) IKKγ ubiquitination by sequestering A20; (4) enhancement of NFκB activity; (5) increased chemokine production; (6) recruitment of neutrophils to infected sites (i.e., where the responding tissue-resident macrophages are located); and (7) Killing of fungi by neutrophils.

We have discussed autophagy-mediated suppression in immune responses (23, 29, 65). Yet, autophagy can enhance antifungal immune responses in early stages of *C. albicans* infection by using mice conditionally lacking *Atg7* in myeloid cells (*Atg7* CKO) (43) (Table 1; Figure 1). The *Atg7* CKO mice showed increased fungal burdens in infected sites as a result of reduced neutrophil recruitment. This was due to reduced production of neutrophil-chemoattractants (CXCL1 and CXCL2) by tissue-resident macrophages at the site of infection in the absence of *Atg7* (43) (Figure 1). Unstimulated tissue-resident macrophages express high levels of A20, an NFκB inhibitor (66–69). After detection of *C. albicans*, autophagy in tissue-resident macrophages sequesters A20 and frees NFκB activation from A20-mediated inhibition. Autophagy-adaptor protein p62 was demonstrated to interact with A20 to carry A20 into LC3-associated cargos (43) (Figure 1). Indeed, the lack of ATG7 or p62 increases the levels of A20, causing reduction in NFκB activity and chemokine production by tissue-resident macrophages (43) (Figure 1). In summary, autophagy appears to function in balancing between inducing antifungal immunity and controlling excessive inflammation.

## Impacts of Autophagy on Adaptive Immunity

Limited amount of autophagy information is available on adaptive immune responses against fungal pathogens, but the involvement of autophagy in shaping adaptive immunity to protect hosts against fungal infections has been suggested. Autophagy enhances survival and functions of T cells (70–72) and B cells (73, 74) in various pathological conditions. Autophagic machinery including ATG5 plays an important role in processing and presenting extracellular microbial antigens in dendritic cells (75). Importantly, LC3-associated cargoes are involved in presenting fungal antigen from *S. cerevisiae* (47). Thus, autophagy appears to control antifungal adaptive immune responses via antigen presentation by DCs and other APCs. It is still not clear whether autophagy in T cells directly controls antifungal immunity.

## Conclusion

Autophagy was initially described as a self-catabolic process, but it is now known to play a critical role in clearance of bacterial, viral, and parasitic pathogens. Although the role of autophagy and ATGs in host defense against fungi had not been made clear, recent studies demonstrated the involvement of autophagy and ATGs in modulating antifungal immunity. LC3-associated cargoes may include fungal spores; but the inclusion cannot be always seen, e.g., when live *Candida* is engulfed. Autophagy and LAP generally protect hosts from majority of fungal infections by inducing immune responses or by controlling excessive inflammation. There are, however, some exceptions that ATGs promote fungal infections. The outcomes of autophagy/LAP in shaping host immune responses appear to greatly vary depending on species of fungi. Interestingly, previous findings suggested that activation of autophagy/LAP by immunosuppressants, such as rapamycin or anakinra (IL-1R antagonist), may result in inducing host resistance against fungal infections. It might be possible that autophagy-related pathways are targeted for new antifungal therapeutics.

## Author Contributions

MK and MLS wrote the manuscript.

## Conflict of Interest Statement

The authors declare that the research was conducted in the absence of any commercial or financial relationships that could be construed as a potential conflict of interest.
